# Effect of alcohol intake on the development of mild cognitive impairment into dementia: A protocol for systematic review and dose–response meta-analysis

**DOI:** 10.1097/MD.0000000000021265

**Published:** 2020-07-17

**Authors:** Lihe Yao, Lijuan Hou, Yongfeng Lao, Wei Luo, Zhenxing Lu, Jun Chen, Yongming Liu

**Affiliations:** a Department of Neurology, The First Hospital of Lanzhou University; b Lanzhou University, First Clinical Medical College Lanzhou; c Evidence-Based Medicine Center of Lanzhou University; d The First Hospital of Lanzhou University; e Lanzhou University, Second Clinical Medical College; f Department of Geriatric, The First Hospital of Lanzhou University, Lanzhou, Gansu, China.

**Keywords:** alcohol, dementia, dose–response, meta-analysis, mild cognitive impairment, protocol

## Abstract

**Objective:**

To assess the dose–response relationship between alcohol consumption and the progression of MCI to dementia.

**Method:**

This study adheres to the Preferred Reporting Items for Systematic Reviews and Meta analysis for Protocols. Chinese Biomedical Literature Database (CBM), PubMed, Cochrane Library, EMBASE will be searched for all relevant published articles, with no restrictions on the year of publication or language. Case–control and cohort studies explored the relationship between alcohol exposure and the incidence of dementia in patients with MCI will be included. Study selection, data collection and assessment of study bias will be conducted independently at each level by a pair of independent reviewers. The Newcastle-Ottawa Scale (NOS) tool will be used for the risk of bias assessment. The methodological quality of systematic review will be based on A measurement Tool to Assess Systematic Review (AMSTAR 2). The Grading of Recommendations Assessment Development and Evaluation (GRADE) system will be used to assess the quality of evidence. Stata 15.0 will be used for general meta-analysis and exploring the dose–response relationship. Piecewise linear regression model and the restricted cubic spline model will be used for nonlinear trend estimation, and the generalized least-square method will be used to estimate the parameters.

**Discussion:**

This dose–response meta-analysis is the first to investigate the dose–effect relationship between alcohol exposure and the incidence of dementia in patients with MCI, providing a comprehensive understanding of the prevention of alcohol-related cognitive impairment.

**Registration:**

The dose–response meta-analysis is registered in the PROSPERO (CRD42019127226) international prospective register of systematic review.

## Abstract 2

Supplemental Digital Content is available in the text

## 1 Introduction

Dementia is a chronic or progressive syndrome in which cognitive function (the ability to process thinking, positioning, understanding et al) deteriorates beyond the expectations of normal aging.^[1,2]^ About 50 million people worldwide suffer from dementia, and by 2050 this number will increase to about 152 million people, which will put a huge burden on families, societies and the medical systems.^[1]^ Mild cognitive impairment (MCI) is a symptom between normal aging and dementia and cannot be diagnosed as dementia.^[3]^ MCI has a high risk of developing dementia.^[4]^ About 10% to 20% of MCI patients become dementia each year,^[5]^ finding risk factors for MCI conversion into dementia has become a way for researchers to consider preventing dementia.^[6,7]^

Alcohol intake is considered a possible risk factor in MCI and dementia.^[8,9]^ A study addressed the dose–response relationship between alcohol intake and mild cognitive impairment.^[10]^ However, the correlation between alcohol intake and MCI conversion to dementia remains controversial. A cohort study found that no more than 15 g of alcohol intake per day reduced the risk of progression to dementia in MCI patients.^[11]^ This is consistent with another cohort study, that is, patients who drink <20 g of alcohol per day and who do not drink have a reduced risk of dementia.^[12]^ However, a cohort study found that drinking more than 1 drink (about 12 g) of alcohol per day accelerates the transition to dementia in MCI patients.^[7]^ Meanwhile, there is no randomized controlled trial or meta-analysis to resolve this controversy or explore specific dose values.

Nowadays, dose–response meta-analysis is considered to be one of the key tools for obtaining high-quality evidence.^[13–15]^ To promote the quality of evidence, we will conduct this dose–response meta-analysis to quantify the relationship between alcohol intake and incidence of dementia in patients with MCI.

## 2 Method

This protocol was registered in the international prospective register of systematic review PROSPERO (CDR42019127226).^[16]^ To improve the quality of our study, This protocol followed the Preferred Reporting Items for Systematic reviews and Meta analysis Protocols (PRISMA-P) guidelines.^[17,18]^ Our systematic review will be conducted and reported following the reporting guidelines provided in the Preferred Reporting Items for Systematic Reviews and Meta analysis statement (PRISMA).^[19]^ No ethical approval and informed consent needed because this is a retrospective study.

### 2.1 Search strategy

A systematic search will be conducted in Chinese Biomedical Literature (CBM), PubMed, Cochrane Library, EMBASE to identify all relevant published articles with no restrictions on year of publication or language.

Previous reviews and meta-analyses as well as the list references of selected studies will be scrutinized to optimize the literature search. The search will be organized according to the three main categories of the population, interventions (exposure), and outcome concepts. The following keywords will be used: preclinical dementia, preclinical Alzheimer's disease, mild cognitive impairment, mild cognitive defect, MCI, alcohol, alcohol blood level, drinking behavior, alcohol consumption, and ethanol. Search will be limited to search fields for titles and abstracts. The strategy adapted to the special characteristics of the database (e.g., the use of medical subject title terms in PubMed). The results of the search will be updated before the final analysis to further identify possible new studies. Details of search strategy of the PubMed database can be found in Supplemental Digital Content (Appendix).

### 2.2 Eligibility criteria

#### 2.2.1 Participants

Patients with MCI are eligible for this study. The diagnostic criteria used for MCI might be various between different research, such as the one proposed by the Mayo Clinic Alzheimer Disease Research Center, including:

1. memory complaint by patient, family, or physician;
2. normal activities of daily living;
3. normal global cognitive function;
4. objective impairment of memory or one other area of cognitive function, as shown by scores >1.5 standard deviations below the age;
5. clinical dementia rating score of 0.5; and
6. not demented,^[20]^ and diagnosis based on the Diagnostic and Statistical Manual of Mental Disorders, Fourth Edition, criteria.^[21]^

There are no specific restrictions on age or sex, no limitations on the comorbidities of patients. Studies focusing on animal and cell culture will be excluded.

#### 2.2.2 Exposures/interventions

Included studies will include at least three levels of alcohol exposure. There is no limit to the unit of alcohol consumption (e.g., quantity unit: drinking volume/week, g/day; frequency unit: times/month). There are no specific restrictions on alcohol types.

#### 2.2.3 Outcomes

The primary outcome will be the incidence of dementia in MCI patients with different alcohol exposure. The diagnosis of dementia was based on the Diagnostic and Statistical Manual of Mental Disorders, fourth edition^[22]^; the National Institute of Neurological and Communicative Disorders and Stroke Alzheimer s Disease and Related Disorders Association criteria for possible and probable AD; and the International Classification of Diseases, 10th revision criteria for VAD and other dementing diseases.^[11]^ For cohort studies, the level-specific hazard ratio (HR) and relative risk (RR) 95% confidence interval (CI) were used to quantify the actual effect, while odds ratio (OR) with 95% CI were often used in case–control studies.

Cohorts (retrospective and prospective cohort studies), case–control studies and nested case–control will be included to explore the effects of alcohol consumption to dementia in MCI patients. Representative, case-only or case–control studies, conference abstracts, reviews, letters and comments will be excluded.

### 2.3 Selection of studies

All investigators will receive appropriate training prior to study screening tasks. The titles and abstracts of the citations retrieved by the literature search will be independently screened by two review authors for potential and relevant studies. The full text is then screened according to eligibility criteria. The reasons for the exclusion of research will be recorded in the full-text screening stage. Any differences regarding inclusion will be resolved through discussions among all review authors. Potential duplicates of included studies will be verified by identifying multiple reports of the same study, overlapping or related studies. For studies with the same sample, we will select the study with the longest follow-up time and the largest sample size. The identification and exclusion process of the study will be described using the PRISMA flow chart.^[19]^

### 2.4 Data collection process

To ensure the mutual understanding of variables, the standardization and unification, reviewers will have a full discussion. Two reviewers will employ a standardized form to extract information independently. The data included:

1. basic information (author, publication year, study design, country setting, and funding information);
2. participant's characteristics (diagnostic criteria, sample size, age, gender, follow-up time, loss of follow-up rate);
3. details of exposure (dosage and frequency of alcohol consumption, method of assessment of alcohol consumption);
4. data on the outcomes (the occurrence of MCI developing into dementia, HR, RR, OR, and 95% CI and covariates adjusted).

The extracted data will be cross-checked by two reviewers to determine consistency and errors. Any disagreement will be resolved through face-to-face discussions or arbitration by third-party reviewers. When the data in the included articles has not been fully reported or missing, the relevant authors will be contacted via their emails or other social platforms, otherwise, we will estimate the data through statistic method, such as we will extract the data from survival curves through Engauge Digitizer (version 9.8).^[23]^

### 2.5 Assessment of study bias

The Newcastle-Ottawa Scale (NOS) tool will be used for the risk of bias assessment of case–control and cohort studies by two review authors independently.^[24,25]^ The scale is given a score of 0 to 9 based on selection (4 items), comparability (1 item), and outcome (3 items). We will represent “low,” “medium,” and “high” quality research with scores of 0–3, 4–6, and 7–9, respectively. Discrepancies in all quality assessments will be resolved after mutual consent and discussion.

### 2.6 Data synthesis and statistical analysis

Stata 15.0 will be used for meta-analysis and exploring the dose–response relationship. The difference was statistically significant when two-tailed *P* < .05. Piecewise linear regression model and restricted cubic spline (RCS) model will be used for nonlinear trend estimation, and the generalized least-square method will be used to estimate the parameters.^[26]^ Cochran's test and the *I*^2^ statistic will be used to measure the heterogeneity of combined studies. The fix-effect model will be used for study merge if the heterogeneity is not significant, otherwise, multivariate meta-analysis will be used to fit the random effect curve and study synthesis. If the nonlinear model is meaningless, a linear model will be applied. If the heterogeneity is large to interpret or the nonlinear model is meaningless, we will abandon the dose–response relationship fitting and present the results in a qualitative description. *I*^2^ > 50% and *P* < .05 will be defined as a significant heterogeneity.

### 2.7 Publication bias and sensitivity analysis

Publication bias will be investigated by visual inspection of the funnel plots and application of Egger's and Begg's tests. Sensitivity analysis will be performed by excluding low/medium methodological quality tests or studies with significant large or small effect values and comparing the results to overall results to evaluate the stability of the results.

### 2.8 Subgroup analysis

Where data is available, the following variables will be used for subgroup analysis:

1. different genders;
2. different types of alcohol intake;
3. different types of study designs (prospective study vs retrospective study);
4. different effect value (HR vs RR/OR).

### 2.9 Confidence of evidence

The methodological quality of systematic review will be based on A measurement Tool to Assess Systematic Reviews (AMSTAR 2).^[27,28]^ The Grading of Recommendations Assessment Development and Evaluation (GRADE) system will be set as a guide to quantify absolute effects and quality of evidence.^[29,30]^ We will assess the quality of evidence in terms of risk, consistency, directness, accuracy, publication bias, and other appropriate areas. The overall strength of the evidence will be judged as high, medium, and low.

## 3 Discussion

This meta-analysis is the first to investigate the dose–response relationship between alcohol intake and dementia in patients with MCI, we will summarize current scientific findings and fill the gap in this field, which may help prevent dementia and provide a reference for national policies on alcohol.

At the same time, the influence of life factors such as alcohol on the disease spectrum has received increasing attention, but existing guidelines often focus on the diagnosis and treatment of specific diseases. The emergence of this broad category of research may create opportunities and challenges for future types of guidance.^[31]^

There may be some possible limitations of this meta-analysis include different units of alcohol consumption that will lead to data integration difficult,^[32,33]^ but we will try to standardize alcohol usage based on the scientific and published method. In addition, dependence on self-reported questionnaires to assess alcohol intake will have measurement bias, however, it may be common in the field category of lifestyle studies. No additional searches are performed for unpublished studies and literature, which means that this review is vulnerable to grey literature bias.

This meta-analysis will systematically explore the dose–response relationship between alcohol intake and dementia in patients with MCI. The results hope to provide high-quality evidence for the current state of research to better drink alcohol and prevent dementia. And this protocol has introduced the methodology details of the target review which will be finished in the future.

## Acknowledgments

We especially thank Peijing Yan, MD, from Evidence-Based Medicine Center of Lanzhou University for her research assistance.

## Author contributions

**Conceptualization:** Yongming Liu, Lihe Yao, Lijuan Hou, Yongfeng Lao.

**Data curation:** Lihe Yao, Lijuan Hou, Yongfeng Lao, Wei Luo, Zhenxing Lu, Jun Chen

**Formal analysis:** Lihe Yao, Lijuan Hou, Yongfeng Lao.

**Methodology:** Wei Luo, Zhenxing Lu, Jun Chen.

**Project administration:** Lihe Yao, Lijuan Hou, Yongfeng Lao.

**Software:** Yongming Liu, Lihe Yao, Lijuan Hou, Yongfeng Lao.

**Supervision:** Yongming Liu.

**Writing – original draft:** Yongming Liu, Lihe Yao, Lijuan Hou, Yongfeng Lao.

**Writing – review & editing:** Lihe Yao, Lijuan Hou, Yongfeng Lao, Wei Luo, Zhenxing Lu, Jun Chen, Yongming Liu.

## Supplementary Material

Supplemental Digital Content
