# Supplementary material for: Effect of alcohol intake on the development of mild cognitive impairment into dementia: A protocol for systematic review and dose–response meta-analysis
Source: Medicine (Baltimore). 2020 Jul 17;99(29):e21265. doi: 10.1097/MD.0000000000021265 (PMC7373509; doi:10.1097/MD.0000000000021265)
Supplement: Supplemental Digital Content [file medi-99-e21265-s001.docx]

Supplementary Appendix 1

**(Effect of alcohol intake on the development of mild cognitive impairment into dementia: A protocol for systematic review and dose–response meta analysis )**

Lihe Yao,MM, Lijuan Hou,MB ,Yongfeng Lao,MB

This Supplementary Appendix shows the retrieval strategy in the pubmeb database.

In this attachment, we only provide retrieval information for the PubMeb database.

**Search strategy**

((((((((((((((((((ethanol[Title/Abstract]) OR alcohol*[Title/Abstract]) OR blood alcohol level[Title/Abstract]) OR blood alcohol content[Title/Abstract]) OR wine*[Title/Abstract]) OR liquor*[Title/Abstract]) OR spirit*[Title/Abstract]) OR beer*[Title/Abstract]) OR beverage*[Title/Abstract]) OR alcohol drinking[Title/Abstract]) OR drinking behavior[Title/Abstract]) OR alcohol consumption[Title/Abstract]) OR alcohol intake[Title/Abstract]) OR drink*[Title/Abstract]) OR drunk*[Title/Abstract])) OR (((("Ethanol"[Mesh]) OR "Blood Alcohol Content"[Mesh]) OR "Alcohol Drinking"[Mesh]) OR "Drinking Behavior"[Mesh]))) AND ((((((((((((((((((((((((((((((((((((((mild cognitive* impairment*[Title/Abstract]) OR mild cognitive* defect*[Title/Abstract]) OR mild cognitive* dysfunction*[Title/Abstract]) OR mild neurocognitive* disorder*[Title/Abstract]) OR mild cognitive* defective*[Title/Abstract]) OR mild cognitive* decline*[Title/Abstract]) OR prodrom* dement*[Title/Abstract]) OR MCI[Title/Abstract]) OR N‐MCI[Title/Abstract]) OR A‐MCI[Title/Abstract]) OR M‐MCI[Title/Abstract]) OR nMCI[Title/Abstract]) OR aMCI[Title/Abstract]) OR mMCI[Title/Abstract]) OR pre-clinical dement*[Title/Abstract]) OR preclinical dement*[Title/Abstract]) OR preclinical Alzheimer*[Title/Abstract]) OR pre‐clinical Alzheimer*[Title/Abstract]) OR preclinical AD[Title/Abstract]) OR pre‐clinical AD[Title/Abstract]) OR age-associated memory impairment*[Title/Abstract]) OR AACI[Title/Abstract]) OR CIND[Title/Abstract]) OR ACMI[Title/Abstract]) OR ARCD[Title/Abstract]) OR SMC[Title/Abstract]) OR CIND[Title/Abstract]) OR BSF[Title/Abstract]) OR AAMI[Title/Abstract]) OR LCD[Title/Abstract]) OR QD[Title/Abstract]) OR AACD[Title/Abstract]) OR MNCD[Title/Abstract]) OR MCD[Title/Abstract]) OR clinical dementia rating scale 0.5[Title/Abstract]) OR CDR 0.5[Title/Abstract])) OR "Cognitive Dysfunction"[Mesh])

**Search Results:** 1091
